# Rapid and accurate detection of multi-target walnut appearance quality based on the lightweight improved YOLOv5s_AMM model

**DOI:** 10.3389/fpls.2023.1247156

**Published:** 2023-11-08

**Authors:** Zicheng Zhan, Lixia Li, Yuhao Lin, Zhiyuan Lv, Hao Zhang, Xiaoqing Li, Fujie Zhang, Yumin Zeng

**Affiliations:** ^1^ Laboratory of Physical Properties of Agricultural Materials, College of Modern Agricultural Engineering, Kunming University of Science and Technology, Kunming, Yunnan, China; ^2^ 69223 Troops, People’s Liberation Army, Aksu, Xinjiang Uygur Autonomous Region, China; ^3^ Project Management Division, Yunnan Provincial Forestry and Grassland Technology Extension Station, Kunming, Yunnan, China

**Keywords:** MobileNetV3, ACMIX, MetaAconC, multi-target, target detection, walnut

## Abstract

**Introduction:**

Nut quality detection is of paramount importance in primary nut processing. When striving to maintain the imperatives of rapid, efficient, and accurate detection, the precision of identifying small-sized nuts can be substantially compromised.

**Methods:**

We introduced an optimized iteration of the YOLOv5s model designed to swiftly and precisely identify both good and bad walnut nuts across multiple targets. The M3-Net network, which is a replacement for the original C3 network in MobileNetV3’s YOLOv5s, reduces the weight of the model. We explored the impact of incorporating the attention mechanism at various positions to enhance model performance. Furthermore, we introduced an attentional convolutional adaptive fusion module (Acmix) within the spatial pyramid pooling layer to improve feature extraction. In addition, we replaced the SiLU activation function in the original Conv module with MetaAconC from the CBM module to enhance feature detection in walnut images across different scales.

**Results:**

In comparative trials, the YOLOv5s_AMM model surpassed the standard detection networks, exhibiting an average detection accuracy (mAP) of 80.78%, an increase of 1.81%, while reducing the model size to 20.9 MB (a compression of 22.88%) and achieving a detection speed of 40.42 frames per second. In multi-target walnut detection across various scales, the enhanced model consistently outperformed its predecessor in terms of accuracy, model size, and detection speed. It notably improves the ability to detect multi-target walnut situations, both large and small, while maintaining the accuracy and efficiency.

**Discussion:**

The results underscored the superiority of the YOLOv5s_AMM model, which achieved the highest average detection accuracy (mAP) of 80.78%, while boasting the smallest model size at 20.9 MB and the highest frame rate of 40.42 FPS. Our optimized network excels in the rapid, efficient, and accurate detection of mixed multi-target dry walnut quality, accommodating lightweight edge devices. This research provides valuable insights for the detection of multi-target good and bad walnuts during the walnut processing stage.

## 1 Introduction

Walnuts (*Juglans* spp.) rank among the world’s top four dried fruits, alongside almonds, cashews, and hazelnuts. Two predominant species of walnuts, common walnuts (*Juglans regia*) and dark-grained walnuts (*Juglans sigillata*), are extensively cultivated globally. *Juglans sigillata*, also known as iron walnut or Yunnan walnut, is an endemic species in Southwest China. It is distinguished by superior seed quality, full kernels, high protein and fat content, and rich nutritional value (Xie et al., 2021). After degreening, rinsing, and drying, the evaluation of the appearance quality of walnuts plays a vital role in bolstering their market competitiveness. Yunnan walnuts, which are characterized by uneven kernel surfaces, non-uniform maturity, varying harvest patterns, and irregular fruit sizes, pose challenges during processing. Existing green walnut peeling machines often yield unsatisfactory results, leaving behind impurities, surface contamination, and an increased susceptibility to breakage (Su et al., 2021). In accordance with the “Walnut Nut Quality Grade” standard GBT20398-2021,^1^ common external defects in walnuts encompass fractured walnut shells, black spots, and insect holes. Black spots on walnut endocarps typically stem from improper peeling, which leaves a residual walnut pericarp on the surface, leading to oxidation and the formation of black spots. In addition to detracting from the appearance quality and grade, these black patches cause mildew due to their moisture-absorbing properties. Furthermore, damaged and insect-infested walnuts expose their kernels to external elements, resulting in rapid deterioration, mould formation, and potentially hazardous substances, such as aflatoxins, due to water infiltration during cleaning. Consequently, there is an urgent need for a rapid and precise method to identify these external defects during walnut production and processing (Li et al., 2019).

Currently, two main approaches are employed to assess produce quality: destructive and non-destructive methods. Destructive methods are utilized to determine the physicochemical or biochemical properties of the produce but require the complete annihilation of the tested specimens, imposing strict technical prerequisites. Although they provide additional phenotypic data, their inherent delay in detection is a drawback. By contrast, non-destructive methods offer advantages such as reduced costs, heightened detection accuracy, and the ability to evaluate produce without damaging it (Arunkumar et al., 2021). Both domestic and international scholars have extensively investigated various non-destructive testing methods for fruits and nuts, including X-ray techniques, acoustic methods (Cobus and van Wijk, 2023), and machine vision approaches (Chakraborty et al., 2023). However, it is worth noting that although these methods excel in detection accuracy, X-ray detection can be expensive, and acoustic methods may be limited to single-target fruit detection, potentially restricting their applicability to primary processing firms.

Deep learning, a non-destructive approach, can swiftly detect issues in one or two phases, offering precise detection and quality control for all types of nuts through computer vision technologies and robotics. The integration of deep learning technology can significantly enhance the production efficiency and quality management within nut processing enterprises by refining the classification and grading processes, automating quality management procedures, and effectively identifying nut defects and abnormalities.

In recent years, researchers have explored a two-stage deep learning approach for fruit and nut detection. For instance, Rika Sustika et al. (2018) investigated the impact of various deep convolutional neural network structures (AlexNet, MobileNet, GoogLeNet, and Xception) on the accuracy of a strawberry grading system (appearance quality detection), with VGGNet demonstrating the highest accuracy (Sustika et al., 2018). Costa et al. (2021) combined machine vision techniques with the Mask-RCNN algorithm (Costa et al., 2021) to detect and semantically segment pecan peel and hull. Fan et al. (2021) proposed an improved rapid R-CNN algorithm (Fan et al., 2021) for the precise detection of green pecans in natural environments. The enhancements included batch normalization, an improved RPN with bilinear interpolation, and the integration of a hybrid loss function. For robot recognition and the picking of walnuts in complex environments, the model achieved an accuracy of 97.71%, a recall rate of 94.58%, an F1 value of 96.12%, and faster detection times. However, these two-stage approaches, which are capable of high accuracy, tend to have slower detection speeds and require lengthy training periods, making them challenging to implement in actual industrial production settings. By contrast, the one-stage approach, represented by the YOLO series algorithm, offers advantages such as fast real-time detection, high accuracy, and robustness. Hao et al. (2022) used an improved YOLOv3 deep learning method for the real-time detection of green walnuts in a natural environment. They pre-trained the model network using the COCO dataset, optimized the performance with data augmentation and K-means clustering, and selected the MobileNetV3 backbone for high accuracy and rapid detection. This approach achieved an average accuracy (mAP) of 86.11% and provided technical support for intelligent orchard management and yield estimation of walnut orchards (Hao et al., 2022). Recognizing the widespread acceptance of the YOLOv5 model as a faster, more accurate, and more efficient target detection model, Yu et al. (2023) proposed an improved walnut kernel impurity detection model based on the YOLOv5 network model. Their model included a small target detection layer, a transformer-encoder module, a convolutional block attention module, and a GhostNet module, leading to enhanced recognition accuracy for small and medium impurities in pecan kernels (Yu et al., 2023). In general, the two-stage target detection algorithm applied to pecans struggles to balance recognition accuracy and detection speed. On the other hand, one-stage algorithm research is tailored to green walnut recognition scenarios. These models and methods cannot be directly applied to the recognition of good- and bad-dried walnuts because of differences in image datasets, such as variations in field orchard backgrounds and occlusion, lighting conditions, or diffractive indices. Additionally, there is limited research on target detection based on deep learning for the classification of dried walnut quality after degreening, washing, and drying during the initial processing stage. Detecting dried walnuts of various target sizes within a wide field of vision presents a challenging task. Therefore, achieving efficient and precise sorting of good and bad dried walnuts using deep learning models has become an urgent matter, significantly impacting the advancement of the entire walnut primary processing industry.

This study introduced an enhanced YOLOv5s_AMM multi-target sorting model tailored for walnuts. (1) The M3-Net network replaced YOLOv5s’ C3 structure with MobileNetV3. This substitution has advantages, such as faster inference, heightened accuracy, reduced memory usage, and improved feature representation. Consequently, it emerges as a superior option for target detection in devices with resource constraints. (2) The model achieved enhanced classification accuracy, adaptive mixture modelling, rapid training and inference, and robustness against noise and outliers by incorporating the novel ACMIX paradigm. ACMIX integrates convolution with self-attentiveness after an SPP (spatial pyramid pooling) layer (He et al., 2015). (3) In the neck layer, the CBM module replaced the activation function of the conventional Conv2d convolution layer with MetaAconC. This substitution results in performance enhancements, adaptive activation, non-linear and smoothing behavior, computational efficiency, and robustness against noise and outliers. Finally, the improved YOLOv5s_AMM detection model, when practically applied to differentiate between good and bad walnuts of various sizes, achieved real-time and efficient classification. This advancement has significant practical value for enhancing walnut detection efficiency, quality, and market competitiveness. This is particularly beneficial to primary processing enterprises aiming to increase the value of their walnut products and contributes to the growth of a more intelligent and integrated walnut industry.

## 2 Materials and methods

### 2.1 Image sample acquisition

**Figure 1:**
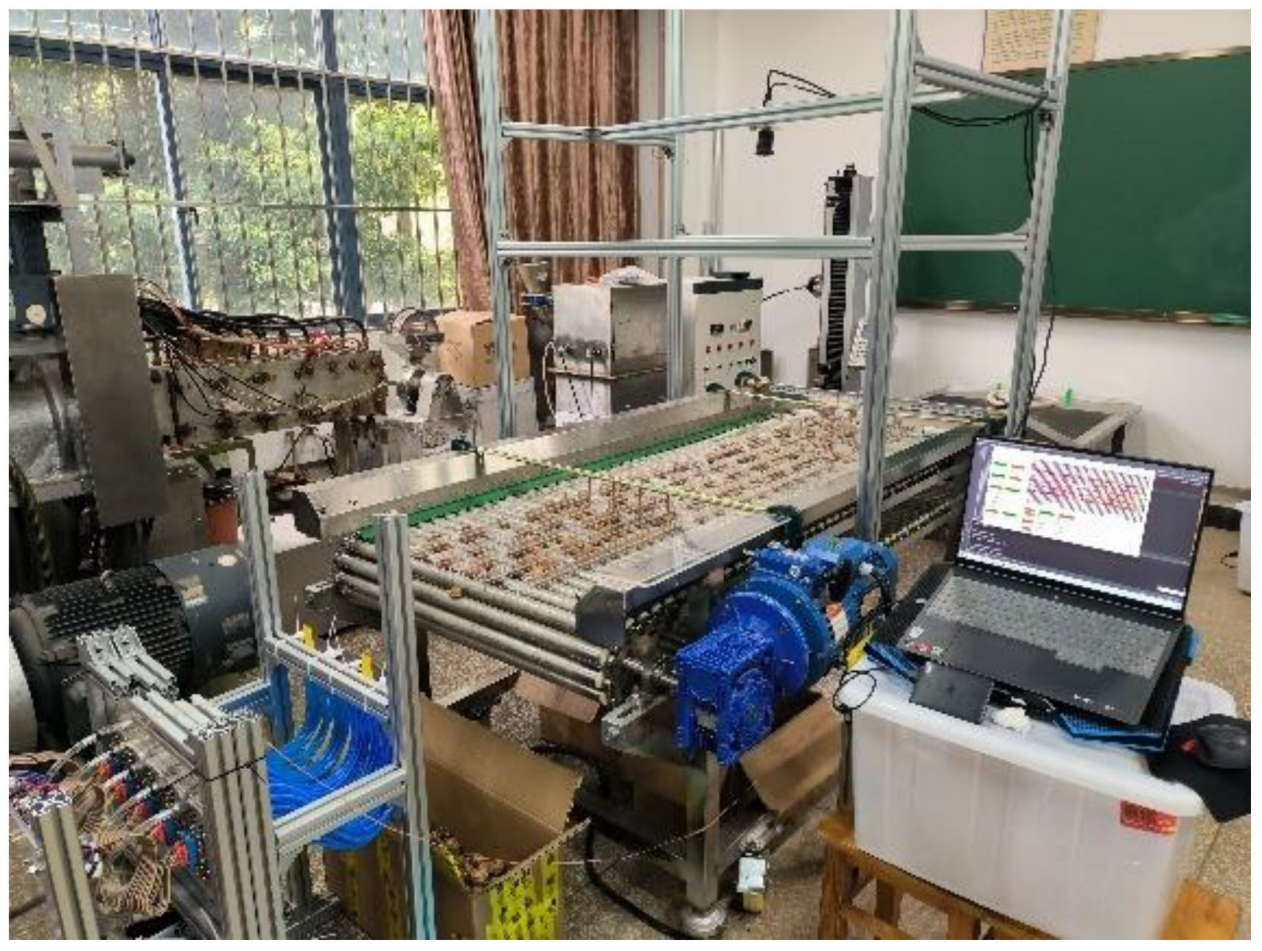
Map of the walnut image acquisition environment.

In this study, walnuts were sourced from Fengqing County, Lincang City, Yunnan Province, China. The RGB images used for the analysis were collected at the Agricultural Material Characterization Laboratory at the Kunming University of Technology. These images were captured from 9:00 a.m. to 6:00 p.m. on December 24–26, 2022. For image acquisition, we employed a Hikvision industrial camera (model MV-CA050-20GC) with a 5-megapixel resolution and a CMOS Gigabit Ethernet industrial surface array camera capable of producing images at a resolution of 2592×2048. The images were saved in the JPEG format. The camera was securely mounted at a height of 195** cm** above the ground and positioned 95** cm** above the surface level using an adjustable aluminium mount. All images were captured under consistent conditions, including the same camera height, uniform light source brightness, consistent background, and roller guide profile phase. The image capture date was December 24, 2022. During the image capture process, we used an exposure time of 4,000 µs and frame rate of 1 in the continuous mode of the camera. This setup allowed us to capture images of walnuts in their natural state, as depicted in **Figure 1**, with the walnuts evenly distributed on the moving part of the profiled roller-wheel guide.

**Figure 2:**
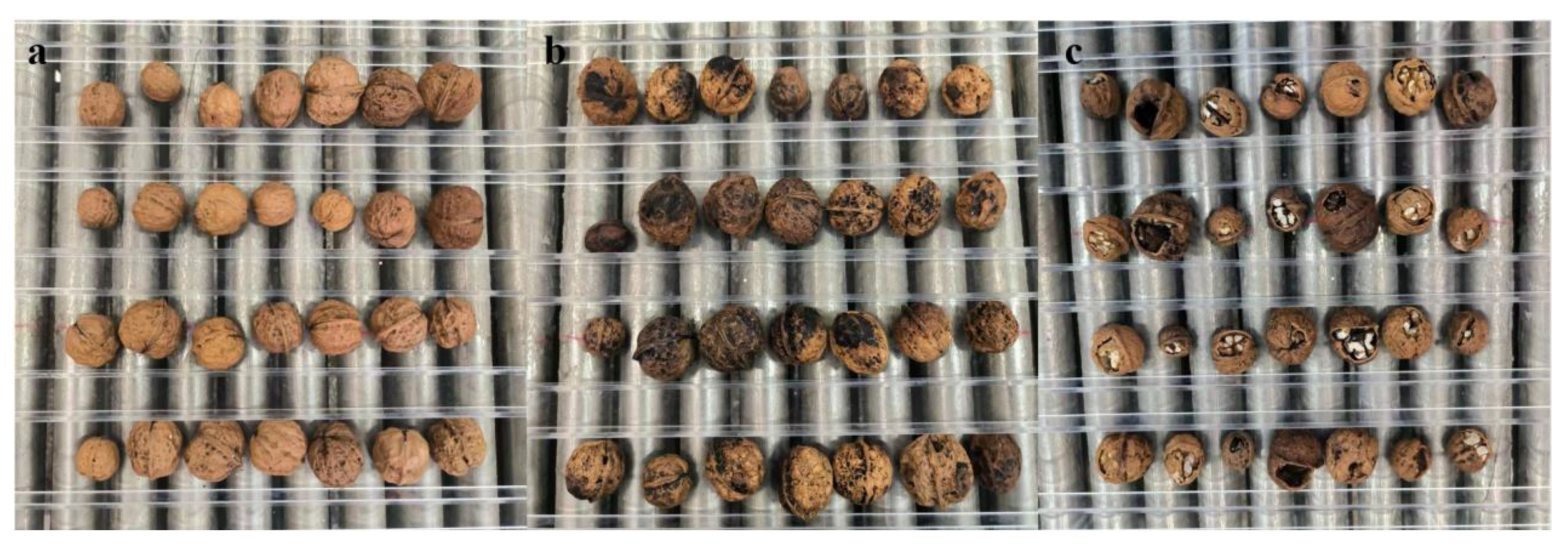
Types of walnuts. **(A–C)** Good walnuts **(A)** and defective walnuts (black spots [**B**] and broken [**C**]).

According to the national standard classification GB/T20398-2021 for walnut quality grades, our evaluation considered various factors such as walnut uniformity, shell integrity, color, and suture line tightness. Based on these criteria, we classified walnuts into two categories: (1) good walnuts (**Figure 2A**), characterized by intact shells and primarily exhibiting a yellow-white color, and (2) bad walnuts (**Figures 2B, C**), including walnuts with black spots (**Figure 2B**) and walnuts with broken shells (**Figure 2C**). In this study, 120 original images with a resolution of 2,592×2,048 were acquired, and multi-target walnuts with excellent walnuts and bad walnuts (black spots and broken) were randomly inserted into this dataset.

### 2.2 Dataset construction

During the data processing phase, we divided the initial dataset of 120 images, each with a resolution of 2,592×2,048, into 2,000 images with a resolution of 640×640. We employed LabelImg software for manual annotation, marking the location boxes, and categorizing walnuts as either good or bad within the original images. This annotation process produced corresponding annotation files. Upon completing the image annotation, we randomly divided the entire dataset into three sets: training, validation, and test. The distribution ratio was 8:1:1, ensuring adequate data for training and model evaluation. In statistical terms, each image in this study contained 5–40 walnuts, resulting in a total of 53,301 labels within the walnut dataset. Among these labels, 25,099 were associated with good walnuts, whereas 28,208 were assigned to bad walnuts. This distribution indicates a reasonably balanced dataset, with a ratio of approximately 0.88 between the two image categories.

**Figure 3:**
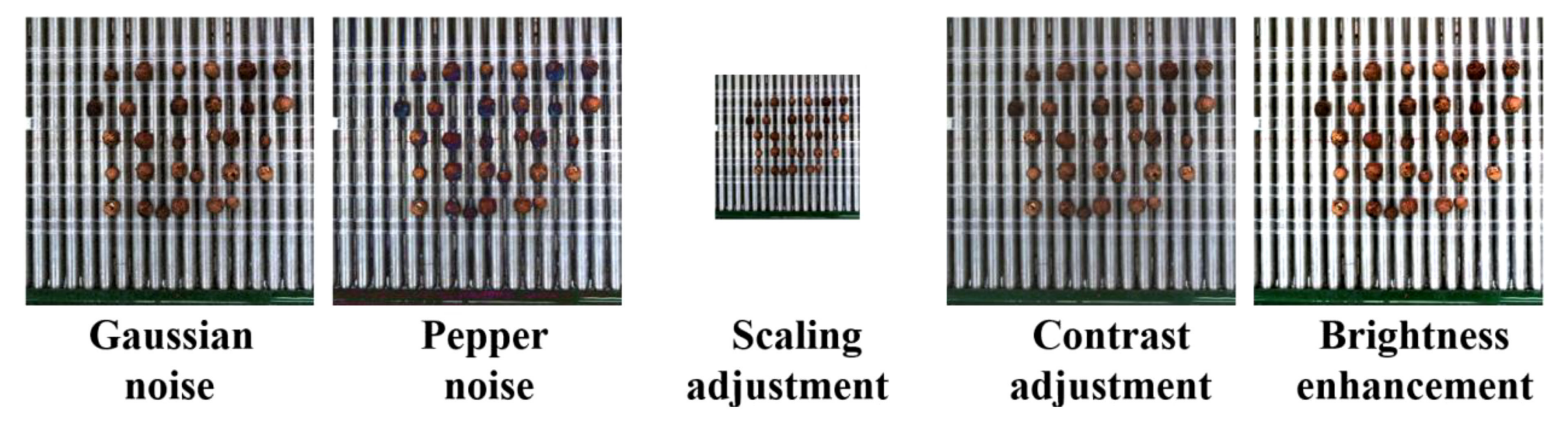
Schematic of the image enhancement of the walnut dataset.

Before commencing model training, we subjected the walnut training set to a combination of offline data enhancement techniques, including contrast adjustment, scaling, luminance modification, pretzel noise, and Gaussian noise (Taylor and Nitschke, 2018). These techniques were applied randomly. As shown in **Figure 3**, They encompassed four specific methods; (1) random contrast enhancement within the range of 0.7 to 1.4 with a gradient of 0.05; (2) random scaling of the training set within the range of 0.5 to 1.5 with a gradient of 0.1; (3) random luminance adjustment for the training set within the range of 0.6 to 1.4; and (4) random modification of the training set’s luminance to either 50–150% of random Gaussian noise or random pretzel noise within the same range. As shown in **Table 1**, these data enhancement procedures resulted in a 25% increase in the number of training sets. Consequently, the walnut dataset contained a total of 2,000 image data entries after data enhancement.

**Table 1:** Classification of the walnut image dataset.

Dataset	Original image	Enhanced image
Training set	1200	1598
Validation set	150	201
Test set	150	201
Total	1500	2000

### 2.3 YOLOv5 network model and optimization structure

#### 2.3.1 YOLOv5 model

The YOLO Network Series provides a rapid and efficient solution for real-time target detection tasks, delivering high accuracy and the capability to identify targets of various sizes. Its versatility extends to a wide range of applications, including autonomous vehicles, robotics, and surveillance systems.

YOLOv5 outperforms YOLOv3 and YOLOv4 in terms of rapid and precise real-time target detection. It achieves this superiority while maintaining a lighter, more efficient, and more easily deployable profile on resource-constrained devices. These improvements encompass several key aspects. (1) Enhanced backbone network: YOLOv5 adopts the CSP-Darknet53 architecture (Bochkovskiy et al., 2020) as its backbone network. This innovation improves feature extraction while reducing the computational cost. (2) Innovative neck layers: The model incorporates SPP and PAN neck layers. These layers combine features from different layers, thereby enhancing both the accuracy and efficiency of the model. (3) Optimized training process: YOLOv5 benefits from the optimized training process. This includes a new hyperparameter search algorithm that efficiently tunes model settings, a novel loss function, improved data augmentation techniques, and AutoAugment, which automatically identifies the optimal hyperparameters. The amalgamation of these enhancements enables YOLOv5 to deliver cutting-edge target detection performance while maintaining a real-time processing speed.

The official code allows for the training of four distinct object detection models with varying depths and widths. In the YOLOv5 series, YOLOv5s serves as the baseline with the smallest depth and width. The other three networks build on this foundation, becoming deeper and more complex. These networks incorporate additional convolutional layers and residual modules in the backbone and employ more channels in the head module to enhance accuracy.

**Table 2** provides a comparison of the accuracy, model size, and detection performance across the four distinct YOLOv5 models. In terms of detection accuracy, YOLOv5s exhibited a slightly lower mAP than YOLOv5m (1.67% lower), YOLOv5l (3.47% lower), and YOLOv5x (3.36% lower). However, when considering the model size, YOLOv5s stood out because of its compact size of 27.1 MB, which was notably smaller than YOLOv5m, offering a reduction of 53.5 MB. This size advantage makes YOLOv5s a cost-effective choice, particularly for deployment on embedded devices, where storage constraints are critical. In terms of detection speed, YOLOv5s outperformed the other models, detecting 7.85 frames per second more than YOLOv5m, 17.13 frames more than YOLOv5l, and 25.97 frames more than YOLOv5x. This superior inference speed position of YOLOv5s is an excellent option for real-time detection scenarios and applications demanding rapid responses. Given the emphasis on low latency and cost-effective deployment for lightweight multi-target kernel peach detection, YOLOv5s presented a compelling proposition with a detection accuracy of 78.97%, a model size of 27 MB, and a detection speed of 47 FPS. It effectively balances accuracy, model size, and inference speed, making it a well-suited base model for further enhancement.

**Table 2:** Comparison of the prediction results from YOLOv5 models.

Model	mAP@0.5 (%)	Parameters	Model size (MB)	FPS
YOLOv5s	78.97	7,276,605	27.1	47.22
YOLOv5m	80.64	7,276,605	80.6	39.37
YOLOv5l	82.44	7,276,605	178	30.09
YOLOv5x	82.33	7,276,605	333	21.25

#### 2.3.2 MobileNetV3: lightweight backbone network

**Figure 4:**
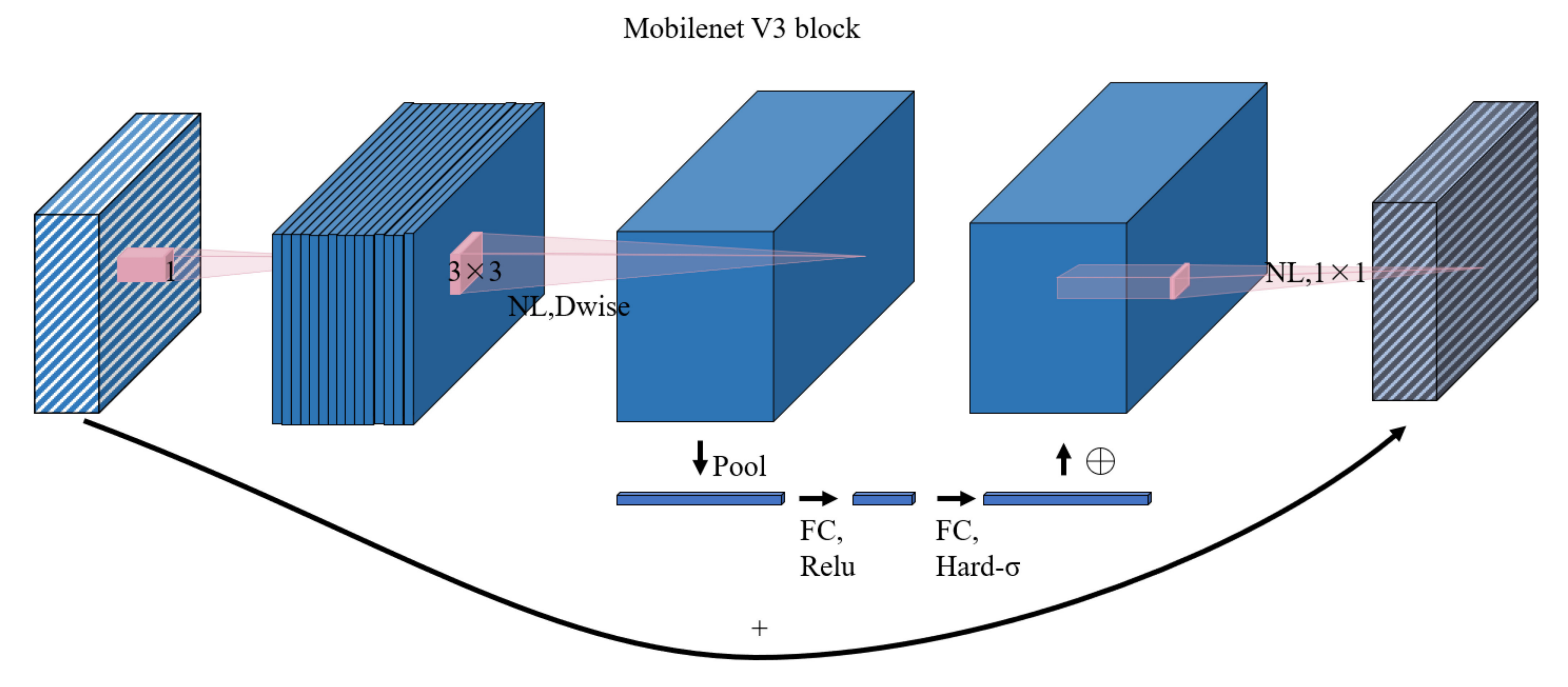
Structural diagram of the MobileNetV3 network.

The concept of a lightweight backbone pertains to neural network architectures optimized for target detection tasks. This optimization involves a reduction in the number of network parameters and layers, while maintaining high accuracy in the target detection tasks. The core objective was to curtail the computational burden and memory requirements of the network. The integration of lightweight backbone networks into target detection models yields substantial advantages, including enhanced computational efficiency, reduced memory demand, accelerated inference speed, and increased robustness. Consequently, they have gained popularity, particularly for resource-constrained applications. In this context, Andrew Howard et al. (2019) proposed the “MobileNetV3” architecture in their research titled “Searching for MobileNetV3” (Howard et al., 2019). Their work demonstrated that MobileNetV3 outperforms alternatives such as ShuffleNet (Bhattacharya et al., 2006) and MobileNetV2 (Sandler et al., 2018) in terms of accuracy, advanced features, and efficient training. This renders MobileNetV3 a versatile and effective option, particularly for resource-constrained devices. **Figure 4** illustrates the structure of MobileNetV3, which includes 1×1 convolutional layers to adjust channel numbers, deep convolutions in high-dimensional spaces, SE attention mechanisms for feature map optimization, and 1×1 convolutional layers for channel number reduction (employing linear activation functions). The network employs residual connections when the step size is 1, and the input and output feature maps have the same shape, whereas in the downsampling stage (step size = 2), the downsampled feature maps are directly output. MobileNetV3’s architectural contributions are primarily grouped into the following categories:

(1) MobileNetV3 leverages deeply separable convolutions and residual blocks to reduce parameters and computations, thereby enhancing the computational efficiency.
(2) Fewer layers are used to minimize the memory requirements and facilitate deployment on resource-constrained devices.
(3) MobileNetV3 incorporates an SE attention mechanism and a hard-swish activation function to support data representation capabilities, while maintaining a modest parameter count and computational load.
(4) The utilization of hybrid precision training and knowledge distillation techniques further enhances the training effectiveness while reducing memory and computational costs.

MobileNetV3 attains state-of-the-art performance across various tasks while retaining its lightweight and efficient nature. This results in substantial reductions in computational and memory costs, rendering it an ideal choice for target detection in resource-constrained devices. This study refined the MobileNetV3 model to enhance its suitability as a lightweight backbone network, thereby achieving higher accuracy and improved network performance.

#### 2.3.3 Acmix: attention-based convolutional hybrid structure

The Acmix architecture (attention-convolution hybrid), introduced in 2021, represents a novel neural network architecture comprising primarily three fundamental modules: an attention module, a convolution module, and a hybrid module. The attention module is responsible for capturing essential features within the input image. Both global and local attention modules are utilized in the Acmix architecture. The global attention module captures the image’s broader contextual information, whereas the local attention module focuses on capturing intricate details within the image. The primary function of the convolution module is the feature extraction from the input image. To achieve this, the Acmix architecture combines the conventional convolutional layers with depth-separable convolutional layers. This integration significantly reduces the computational cost and memory requirements of the network, thereby enhancing its overall efficiency. The hybrid module serves as the nexus where the features extracted by the attention module and convolution module converge and interact. In this context, the Acmix architecture uses both global and local hybrid modules. The global hybrid module harmonizes characteristics from the global attention module and convolutional module, whereas the local hybrid module fuses attributes from the local attention module and convolutional module.

**Figure 5:**
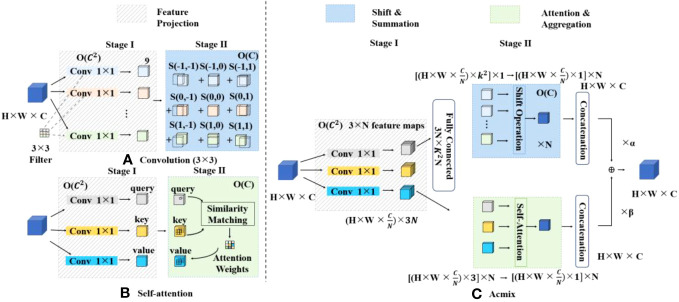
Structure of the hybrid module network in Acmix.

**Figure 5** illustrates the hybrid module proposed by Acmix. The left diagram shows the flowchart of the conventional convolution and self-attention module. (a) The output of the 3×3 convolutional layer can be decomposed into a summation of shifted feature maps. Each of these feature maps was generated by applying a 1×1 convolution with kernel weights at specific positions, denoted by s(x,y). (b) The self-attention process involves projecting the input feature maps into queries, keys, and values, followed by 1×1 convolution. The attention weights computed through the query-key interaction were used to aggregate the values. Conversely, the diagram on the right delineates the pipeline of our module. (c) Acmix operates in two stages. In stage one, the input feature map underwent projection using three 1×1 convolutions. Stage two employs intermediate features using two examples. The characteristics extracted from both paths are fused to generate the final output. The computational complexity of each operation block is shown in the upper corner (Pan et al., 2022). The Acmix architecture has demonstrated state-of-the-art performance across various benchmark datasets for image classification tasks, while maintaining a lightweight and efficient design. The attention and hybrid modules within Acmix are strategically designed to capture both global and local features within walnut images, with a particular emphasis on identifying black spots and damaged areas on walnuts. Consequently, the Acmix module is introduced after the SPP module during the feature fusion phase of the improved model to enhance its performance, particularly on intricate datasets.

#### 2.3.4 MetaAconC activation function


(1) $$\text{MetaAconC}=(p_{1}-p_{2})X\delta \left[\beta_{c}(p_{1}-p_{2})X\right]+p_{2}X,\beta_{c}=\delta W_{1}W_{2}\sum_{h=1}^{H}\sum_{w=1}^{W}X_{c,h,w}$$

In Eq. (1), X represents the input feature map, where X (c,h,w) denotes the feature input with dimensions of C × H × W. W1 and W2 represent the computed weights; p1 and p2 represent adjustable learning parameters; β signifies the adaptive function; and δ represents the sigmoid activation function (Nan et al., 2023). MetaAconC (Ma et al., 2021) is a novel activation function proposed in 2021 to address the limitations of conventional activation functions. This is achieved by combining the Meta-AC and CAN functions, which are known to be vulnerable to the gradient vanishing problem and can lead to neuron inactivity. The Meta-AC function is capable of concatenating multiple activation functions and adapting to the specific input data distribution. The CAN function non-linearly transforms the output generated by the Meta-AC function, enabling it to capture more intricate and abstract features. Empirical evidence has shown the superiority of the MetaAconC activation function over traditional alternatives, such as ReLU and sigmoid. It offers distinct advantages, including adaptivity, computational efficiency, and robustness against noise and outliers. These attributes were substantiated in subsequent ablation experiments. In the context of our enhanced model, the original SiLU activation function was replaced by the MetaAconC activation function. The experimental data underscore its suitability for walnut image detection.

#### 2.3.5 Improved Yolov5s network structure

**Figure 6:**
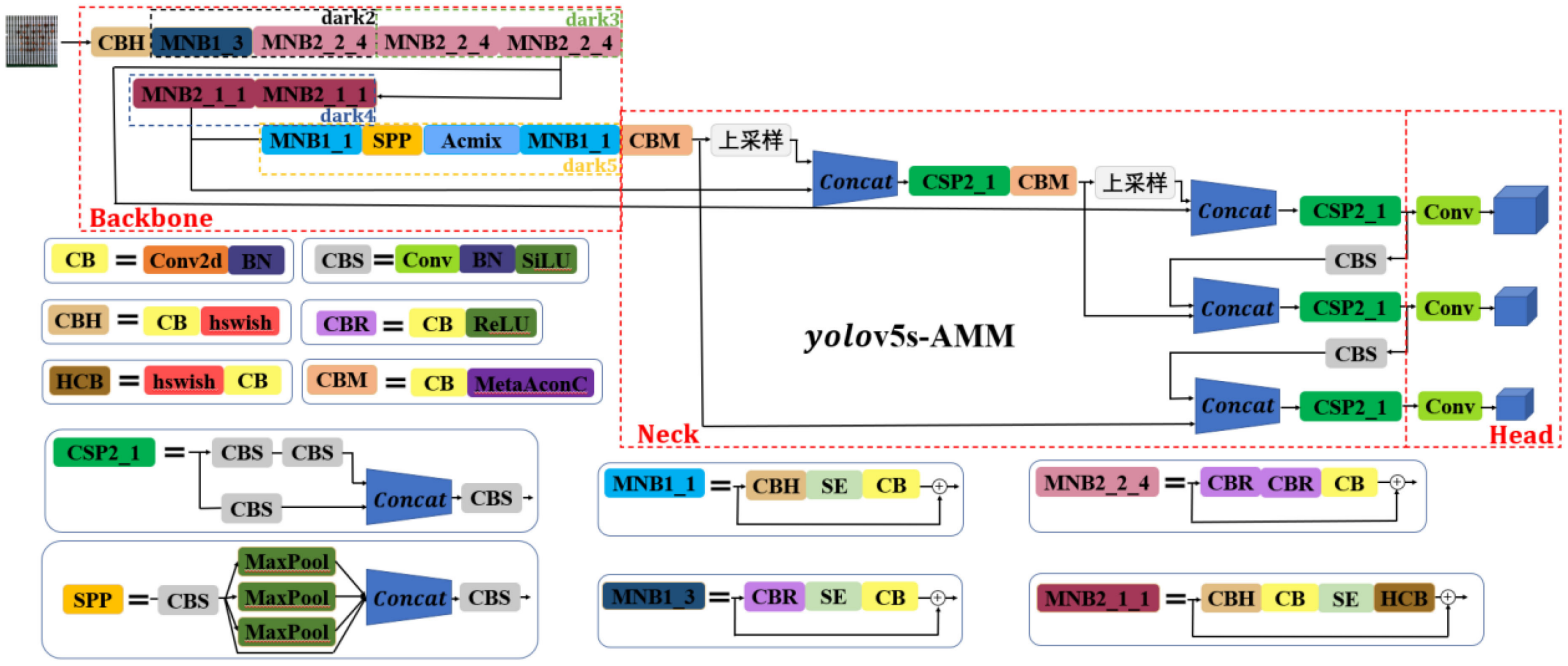
Overall network structure of the improved YOLOv5s-AMM model.

In this study, we built upon the architecture of YOLOv5s, version 5.0, as the foundation for model improvement. The objective was to address issues related to accuracy, model size, and detection speed to develop a more appropriate model for the detection of good and bad walnut fruit targets during the primary processing stage. The overall enhanced network structure is shown in **Figure 6**. In this model refinement, we opted to replace the original focus layer with CBH and the C3 backbone network structure with MobileNetV3 from the M3-Net network. This alteration was made with the aim of reducing the model size and ensuring a lightweight and efficient design. Furthermore, we introduced the attention convolutional hybrid (Acmix) structure into the neck layer. This addition reduced the computational cost and memory requirements of the network. The attention and hybrid modules within the Acmix architecture are strategically designed to capture both global and local image features, thereby enhancing the model’s performance on complex datasets. Finally, we replaced the two Conv2d modules in the neck layer with the CBM modules. In addition, the SiLU activation function found in the original Conv layer was substituted with the MetaAconC activation function. This adjustment is implemented to improve the input-specific data distribution for tuning, ultimately enhancing the feature detection across different image scales.

**Table 3** provides an overview of the replacement lightweight backbone network used in this study, with a primary focus on the incorporation of the M3-Net network to construct the backbone network of the enhanced model. **Table 3** presents detailed information on various parameters and components. Specifically, “Input” represents the features of the input layer feature matrix; “#Out” represents the number of channels in the output layer feature matrix; “S” represents the step size of the DW convolution; “exp size” represents the size of the first up-convolution; “SE” indicates whether the attention mechanism is employed; “NL” represents the activation function used; “HS” represents the hard-swish activation function; and “RE” represents the ReLU activation function. Within the modified backbone layer of M3-Net, there are primarily four types of MobileNet_Block:

MNB1_1: CBH + SE attention mechanism + CB
MNB1_3: CBR + SE attention mechanism + CB
MNB2_1_1: CBH + CB + SE attention mechanism + HCB
MNB2_2_4: CBR + CBR + CB.

**Table 3:** Backbone network with the improved model.

Input	Operator	Exp size	#out	SE	NL	S
640^2^×32	Conv_Bn_Hswish	–	64	–	–	1
320^2^×64	MobileNet_Block,3×3	64	64	√	RE	2
160^2^×64	MobileNet_Block,3×3	384	128	–	RE	2
160^2^×128	MobileNet_Block,3×3	448	128	–	RE	1
80^2^×128	MobileNet_Block,5×5	512	256	√	HS	2
40^2^×256	MobileNet_Block,5×5	960	256	√	HS	2
40^2^×256	MobileNet_Block,5×5	512	512	√	HS	1
20^2^×512	MobileNet_Block,5×5	512	512	√	HS	2
20^2^×512	SPP	–	512	–	–	–
20^2^×256	Acmix	–	512	–	–	1
20^2^×256	MobileNet_Block,5×5	512	256	√	HS	1

First, the original focus layer was replaced with the Conv_Bn_Bswish layer, resulting in improved model accuracy, accelerated inference, and architectural simplification. Moreover, the Conv and C3 components of the original dark2 layer were replaced with MNB1_3 and MNB2_2_4, while the Conv and C3 components of the dark3 layer were replaced with two consecutive MNB2_2_4 structures. This replacement strategy employed MobileNetV3_Block to construct a lightweight backbone network, which not only enhanced the accuracy in identifying the walnut dataset but also boosted the network performance efficiency, facilitating faster convergence and superior generalization effects. Subsequently, we introduced the Acmix structure after applying the SPP structure to the output of the final layer. This involved a combination of standard convolutional layers and deeply separable convolutional layers to reduce the neocortex size. Consequently, network performance efficiency was further enhanced, leading to faster convergence. As a result, when the input image size was set to 640×640, the improved backbone network generated output feature maps with dimensions of (20 × 20×1,024), (40×40×512), and (80×80×256). The role of the neck is to integrate the walnut characteristics extracted from the backbone into a format suitable for object detection. This component plays a pivotal role in improving the accuracy of the walnut target detection model by capturing the walnut features at various scales and combining them effectively. This enhances the model’s ability to detect objects of varying sizes and aspect ratios. In addition, when replacing the corresponding conv2d module with a CBM module after the (20×20×1024) and (40×40×512) walnut feature maps, the MetaAconC activation function in the CBM module surpasses the performance of the sigmoid function. It is not only adaptive but also capable of learning based on the specific walnut data distribution. This is in contrast with the traditional sigmoid function, which remains fixed and unalterable during the training process. The MetaAconC function offers high computational efficiency, leading to an improved detection performance for walnut images at various scales. Furthermore, it reduces the computational load and memory usage of the network, resulting in shorter inference times and reduced hardware requirements. Finally, the head layer produced output feature maps with dimensions of 80×80×(3×(num_classes+5)), 40×40×(3×(num_classes+5)), and 20×20×(3×(num_classes+5)). Here, “num_classes” denotes the number of detected walnut object classes in the training network, and “3” denotes the number of anchor boxes used for walnut object detection within each grid cell.

#### 2.3.6 Training the multi-target detection model for walnuts (good and bad fruits)

To impart the model with more relevant and informative features, the initial image was segmented into 640×640 pixels, aligned with the model’s input image size of 640×640 pixels. Building on this foundation, the model was enhanced using the proposed improvement methodology. Subsequently, the labelled walnut dataset was employed for training within the PyTorch deep learning framework, whereas the validation dataset served as a means to evaluate the effectiveness and performance of the model training process.

**Table 4** lists the experimental settings used in this study. Initially, the dataset containing annotations in the VOC format was converted into a format compatible with the YOLOv5 model. Subsequently, the parameters governing the training procedure were configured meticulously. The enhanced YOLOv5s detection network was then subjected to training with an initial learning rate of 0.01, eta_min at 1 × 10^-4^, last_epoch at −1, momentum parameter at 0.937, delay parameter at 5 × 10^-3^, batch size at 8, and T_max at 250. The optimization during the training procedure was executed using an SGD optimizer. Multi-threaded model training harnessed the computational power of the four processors, whereas the cosine annealing learning rate was dynamically updated for optimization during training. Furthermore, four offline enhancement techniques, including contrast adjustment, scaling, luminance modification, and the introduction of pepper and Gaussian noise to the walnut image data, enrich the contextual information for detecting walnut objects. These augmentations enhance the perception of distinguishing between good and bad walnut features, thereby bolstering the model’s robustness and generalization capabilities. Notably, the data augmentation network required approximately 6 h and 52 min to complete the training process.

**Table 4:** Experimental settings for this study.

Name	Value
CPU	AMD Ryzen 9 5900HX with Radeon Graphics octa-core
Memory	32 GB
Storage SSD	1024GB
Graphics card	NVIDIA GeForce RTX 3080
Graphics memory	16GB
Operating System	Windows11
CUDA version	11.6
PyTorch version	1.8.0

The entire training process was segmented into two distinct phases, namely, the “freezing phase” and the “thawing phase”, in alignment with the underlying model structure. During the freezing phase, the spine of the model remained unaltered and was held constant. No modifications were made to the ad hoc extraction network. During this phase, the focus was on training the weight parameters of the prediction network until they reached a state of saturation and convergence. Subsequently, the model entered the thawing phase, wherein the core of the model was no longer constrained and the weight parameters of the feature extraction network were subjected to training to optimize the entire set of network weights.

The loss curve in target detection serves as a crucial indicator of the training progress of the model by monitoring the value of the LOSS function. This function is a mathematical construct that quantifies the disparity between the model’s predicted output, given an input image, and the actual output (i.e., the true value). Within the context of YOLOv5, the loss function comprises several integral components, including localization, confidence, and class loss. The localization loss is responsible for assessing the precision with which the model predicts the coordinates of bounding boxes around objects within an image, whereas the confidence loss quantifies the level of confidence in the model’s prediction. The class loss measures the capacity of a model to classify images.

The loss curve indicates the model’s learning progress in generating accurate predictions after being trained on the dataset. As the model acquires knowledge from the training data, the loss progressively diminishes. The objective of training is to minimize this loss, which indicates that the model makes accurate predictions on the training data.

**Figure 7:**
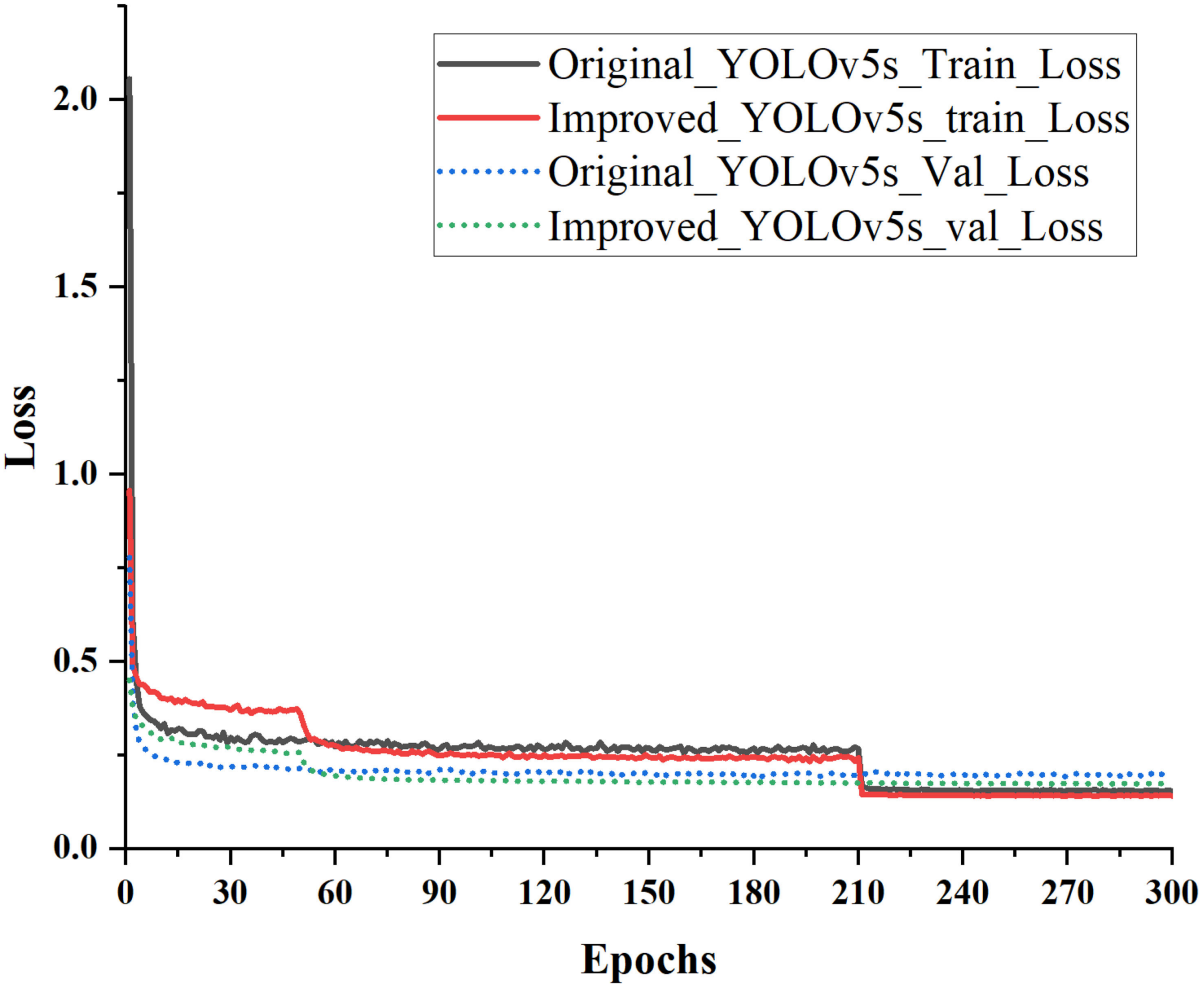
Comparison of epochs trained using original and improved models.

As depicted in **Figure 7**, the initial model observed a notable reduction in the loss value during the fifth iteration. Subsequently, from the fourth to the tenth iteration, the loss value stabilized, hovering at approximately 0.32. Notably, there was no discernible alteration in the thawing stage, even as the model progressed to the 50th iteration.

Following the unfreezing of the model, the loss decreased notably between the 50th and 53rd iterations,.from 0.36 to 0.29. Moreover, between the 60th and 210th iterations, the loss remained consistently lower than that of the original model. Subsequently, a comparison between the original and improved models’ loss values from iteration 210 onwards revealed that the improved model exhibited a swifter decline in losses between iterations 210 and 300. Ultimately, the loss values of the improved model stabilized at approximately 0.142, a reduction of 0.01 points compared with the original model. These results highlight the superior performance of the enhanced model in distinguishing between good and bad walnuts during the convergence.

## 3 Results

### 3.1 Model evaluation indicators

To conduct a thorough evaluation of the model’s performance on multi-target walnut images, we employed eight widely accepted evaluation metrics that are commonly used in classical target detection algorithms. These metrics included precision (P), recall (R), F1 score, average precision (AP), average accuracy (mAP), network parameters, model size, and detection speed. Throughout the experimental period, an IoU value of 0.50 was used. To assess real-time detection performance, this study employed frames per second (FPS) as the key metric. A higher FPS indicates a higher model detection rate. Equations (1)–(5) illustrate the specific formulas for calculating P, R, F1, AP, and mAP.


(2) $$\text{P}=\frac{\text{TP}}{\text{TP}+\text{FP}}$$

(3) $$\text{R}=\frac{\text{TP}}{\text{TP}+\text{FN}}$$

(4) $$\text{F}1-\text{ score }=\frac{2\text{PR}}{\text{P}+\text{R}}$$

(5) $$\text{AP}\left(\text{k}\right)=\int_{0}^{1}\text{P}\left(\text{R}\right)\text{dR}$$

(6) $$\text{mAP}=\frac{\sum_{1}^{N}\text{AP}(\text{k})}{\text{N}}$$

where TP represents the number of correctly identified walnuts (true positives); FP represents the instances in which the classifier incorrectly predicted positive samples among the actual negative samples (indicating the number of false negative samples); TN represents the number of correctly identified negative samples; and FN represents the number of negative samples that were incorrectly predicted by the classifier.

The F1 value serves as a comprehensive measure of the overall accuracy of the detection model and is calculated as the average sum of precision and recall. AP represents a measure of the precision and recall trade-off for a given detection model by calculating the area under the recall curve. Higher AP values indicate a better performance. In equation (6), “N” represents the number of object categories, “AP (k)” is the average precision for a specific category (in this study, k=2), and “∑” signifies the sum across all categories. mAP consolidates accuracy and recall across multiple object categories, offering a global assessment of the object detection model’s performance. The scores range from 0 to 1, with higher scores indicating superior performance. Given the need to evaluate an integrated object detection network with multiple object categories and the superiority of the mAP over the F1 score, we chose to use the mAP score for our assessment.

### 3.2 Experimental results

**Table 5** presents the evaluation results of the enhanced model using the 201-objective walnut test dataset. The empirical findings revealed that the improved YOLOv5s model achieved an overall mAP of 80.78% on the test dataset. Additionally, it attained an F1 score of 0.77, a model size of 20.90 MB, and an average detection rate of 40.42 frames per second, thus satisfying the real-time detection requirements. The precision-recall gap across each category ranged from 2.62 to 4.46%. Furthermore, the cumulative mAP for both excellent and bad walnuts was 80.78%. In summary, the enhanced model proposed in this study for the detection of good and bad walnut fruits demonstrates superior accuracy, minimal computational overhead, and rapid inference capabilities.

**Table 5:** Experimental results.

Class	P/%	R/%	mAP@0.5 (%)	F1-score	Model size (MB)	FPS
Good	75.11	70.65	86.79	0.73	20.9	40.42
Bad	82.13	79.51	74.78	0.81		
All	78.62	75.08	80.78	0.77		

### 3.3 Effect of the detection performance of attention mechanisms at various positions

**Figure 8:**
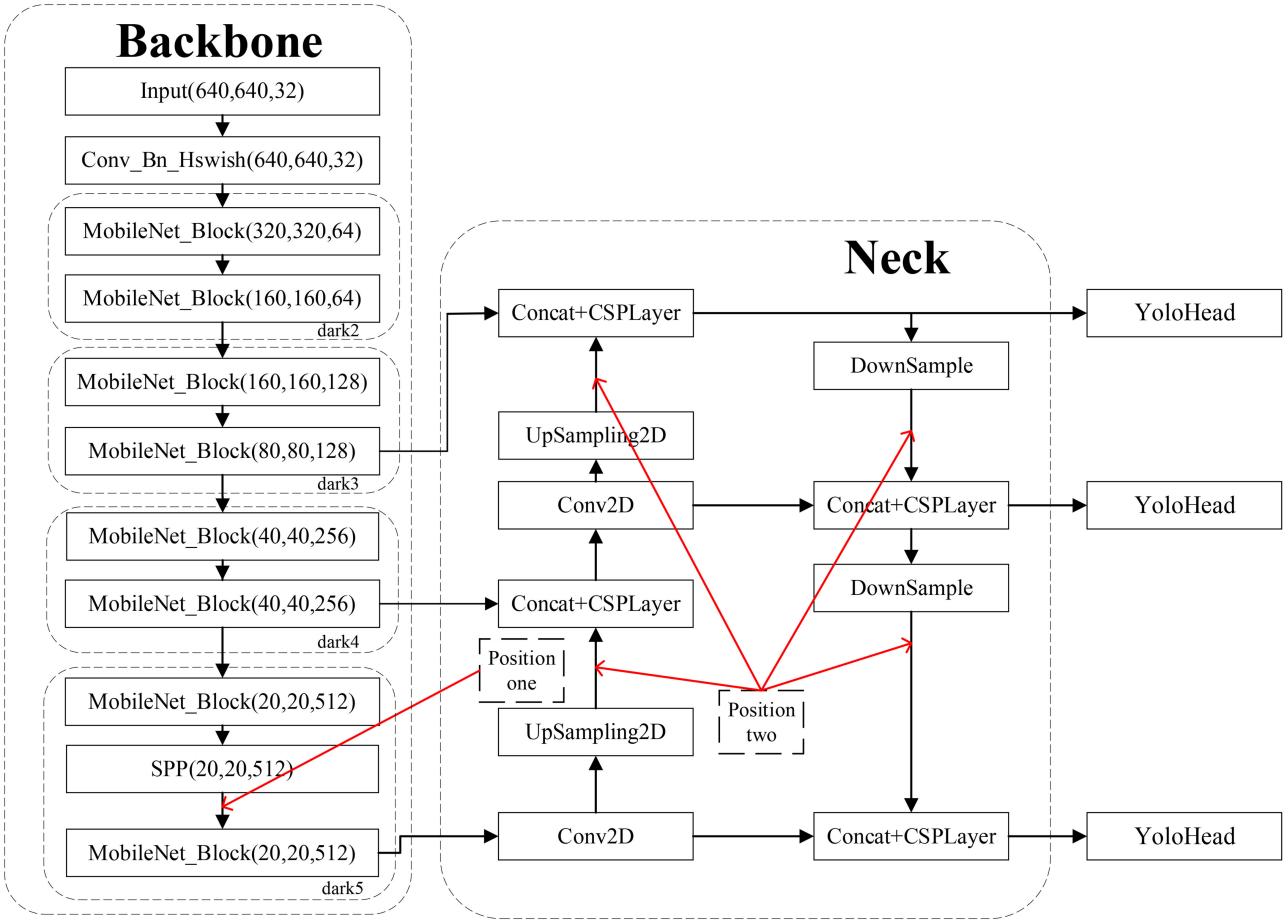
Integration of attention mechanisms at different locations in the original YOlOv5s model.

In the context of the original YOLOv5s network, we introduced the attentional convolutional hybrid Acmix module into both the backbone and neck layers, as depicted in **Figure 8**, to explore the impact of integrating the attentional mechanism at different locations within the model. Specifically, after pyramidal pooling in the SPP space, the final dark5 module at location 1 in the backbone layer incorporated the Acmix module. The Acmix module received the output feature maps from the SPP layer and calculated the channel weights, which were subsequently applied to the input feature maps. This process emphasized significant regions while suppressing insignificant ones. Additionally, at position 2, located after each upsampling and downsampling operation in the neck layer, another Acmix module was applied.

**Table 5** presents the results of comparing the effects of adding the attention mechanism at various locations. Notably, the addition of Acmix at location 1 improved the model’s mAP by 1.38%, increased the model size by a mere 3.2 MB, and reduced the detection speed by only 4.08 frames per second. However, when added at location 2, the model’s mAP experienced a marginal decrease of 0.07%, accompanied by a more significant reduction in detection speed by 18.83 frames per second. This suggests that the Acmix attention mechanism may not be universally applicable to all layers. The reason for this discrepancy lies in the fact that introducing too many attention mechanisms in location 2 of the neck layer may diminish the model’s mAP. In addition, the excessive incorporation of attention mechanisms at location 2 introduces a surplus of additional parameters, potentially resulting in network overfitting and deterioration in network performance. The most pronounced enhancement was observed when Acmix was added at location 1, particularly when compared with location 2, or when both locations received attention mechanisms. This is due to the multifaceted scale features generated by SPP, which aid in the detection of targets of varying sizes but have weaker interrelationships. Adding the Acmix attention mechanism after SPP explicitly constructs relationships between features of different scales, enabling the network to better leverage these features and subsequently enhance its performance. Additionally, because SPP generates a surplus of features, some of which may be irrelevant, the Acmix attention mechanism effectively filters out these irrelevant features, focusing on the most pertinent features to reduce feature redundancy. Furthermore, it enables the learning of novel feature expressions based on the features generated by SPP, thereby improving the overall feature representation. Incorporating Acmix attention at position 1 effectively compensated for the limitations of SPP and resulted in more potent feature expressions.

### 3.4 Effect of various attention mechanisms on detection performance

In the context of the original YOLOv5s model, we introduced various attention mechanisms from position 1 in **Figure 8** to investigate their impact on the performance of the target detection model. As shown in **Table 6**, the addition of the Acmix attention mechanism exhibited the most substantial improvement in the mAP performance compared with the original model, achieving a notable increase of 1.38%. By contrast, the ECA, CBAM, and SE attention mechanisms displayed comparatively less improvement in the mAP performance. This observation underscores that merely applying an attention mechanism after SPP does not inherently improve model accuracy; rather, its effectiveness depends on the structural properties of the network and characteristics of the recognition objects. In this study, we selected the Acmix attention mechanism due to its superior performance. Acmix possesses the unique capability of dynamically adjusting channel weights by calculating the global attention map for each channel. By contrast, ECA (Wang et al., 2023), CBAM (Woo et al., 2018), and SE (Hu et al., 2018) employ fixed channel weights. This dynamic adjustment allows Acmix to highlight critical channel information more precisely, effectively enhancing the features of the walnut images. Additionally, Acmix can concurrently capture a more comprehensive spatial-frequency feature representation by combining location and channel attention information. The introduction of location attention further promotes channel attention, thereby enhancing the extraction of pertinent location features from walnut images. Conversely, ECA, CBAM, and SE consider only a single type of attention, whether it is location, position, or channel. Moreover, although model size and inference speed are crucial considerations, accuracy remains paramount. As depicted in **Table 6**, the additional model burden introduced by Acmix was a mere 3.2 MB, and the reduction in FPS was a modest 4.08 frames per second. The increase in the parameters, although present, does not overly complicate the model. Given that the Acmix hybrid mechanism comprehensively captures information, combining both spatial and channel contextual insights and significantly enhancing mAP, the slight reduction in computational efficiency and detection speed remains acceptable. Although the task of simultaneously increasing detection accuracy while maintaining model efficiency is inherently challenging, the experimental findings suggest that incorporating attention mechanisms can mitigate this challenge to some extent. For instance, **Table 6** illustrates that the inclusion of attention modules (e.g., Acmix, ECA, etc.) can effectively improve the mAP with minimal expansion in model size. Among these mechanisms, Acmix attention stands out by achieving the best accuracy improvement, driven by its ability to integrate spatial and channel contextual information. Considering all factors related to model accuracy, size, and detection speed, the Acmix attention mechanism emerges as the optimal choice, striking an excellent balance between accuracy enhancement, model size, and detection speed.

**Table 6:** Comparison of target detection model capabilities with the addition of various focus mechanisms.

Attention mechanisms	mAP0.5 (%)	Parameters	Model size (MB)	FPS
None	78.97	7,276,605	27.1	47.22
ECA	79.15	7,276,866	27.1	50.95
CBAM	78.90	7,278,751	27.1	51.88
SE	79.69	7,277,629	27.1	51.03
Acmix	80.35	8,106,537	30.3	43.14

### 3.5 Enhancing detection performance for varied target sizes

**Figure 9:**
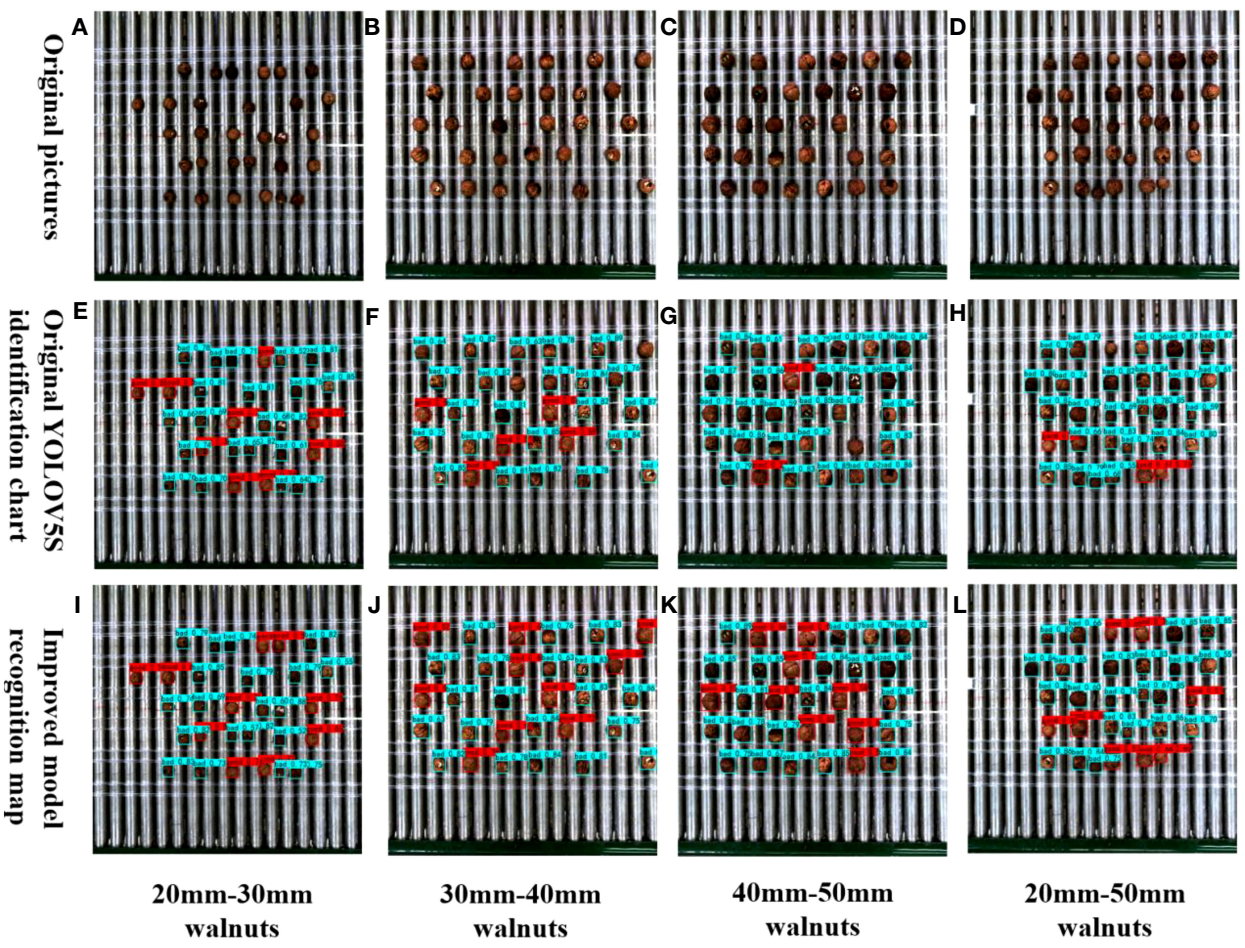
Comparison between the original and improved models for detecting various sizes of mixed walnuts.

The classification of good and bad walnuts was notably affected by the degreening and drying process. Walnuts typically sold fall within the size range of 20–50 mm, and the initial processing of walnuts after degreening and drying significantly influences their classification. It is particularly crucial to ensure a sufficiently large field of view in the context of multi-target walnuts to improve grading efficiency. Additionally, evaluating the recognition performance of the model for multi-target walnuts in an actual mixed forward conveying scenario represents a rigorous test of its capabilities. To investigate the performance of the detection model for multi-target walnuts of varying sizes in a mixed scene, as illustrated in **Figure 9**, we employed 30 small target walnuts measuring 20–30 mm, 30 medium target walnuts ranging from 30–40 mm, and 30 large target walnuts spanning 40–50 mm. Each size category included 10 good walnuts and 20 bad walnuts (10 with black spots and 10 broken fruits). Additionally, we incorporated 30 walnuts ranging in size from 20 to 50 mm (10 walnuts per size), featuring 3 good walnuts and 7 bad walnuts in each size (4 with black spots and 3 broken walnuts). Comparing the small-target detection results in **Figures 9E, I**, it becomes evident that the improved model identified good walnuts within the small-target category. This improvement can be attributed to the replaced MobileNetV3 module, which effectively captures multiscale information through depth-separable convolution, enhancing the recognition of key features, such as the morphology of small target walnuts. Upon comparing the target images in **Figures 9F, J**, the large target images in **Figures 9G, K**, and the mixed target detection images in **Figures 9H, L**, it becomes apparent that the original model struggled to identify good walnuts, particularly in the case of multiple targets, in which simultaneous identification was problematic. By contrast, the improved model adeptly identified each individual walnut, significantly enhancing the detection accuracy of healthy fruits. This improvement can be attributed to the addition of the Acmix attention mechanism after the SPP layer, which effectively captures spatial feature information related to walnut fruit shape and surface texture across multiple scales. Meanwhile, the MetaAcon activation function is more effective at expressing non-linear features than SiLU, enabling the extraction of complex features, such as walnut fruit color, and aids in the identification of walnuts of varying sizes. Therefore, the improved YOLOv5s_AMM model demonstrates enhanced recognition performance in the mixed recognition of multi-target walnuts at different scales, maintaining a high recognition count and rate. Although a single false detection occurred in the small-target scene, the overall false detection rate remained below 3%. Future research efforts will address this issue by refining the structure of the model to detect small-sized targets.

### 3.6 Ablation experiments

Ablation experiments were performed on the original YOLOv5s model to assess the impact of various enhancement strategies on the detection performance. All improvement procedures were trained and validated using identical training and validation datasets, and evaluated using the same test dataset. The experimental results are presented in **Table 7**. The original YOLOv5s model achieved an mAP of 78.97% based on 201 test images. It featured a parameter count of 7,276,605, model size of 27.1 MB, and FPS of 47.22 frames per second, as detailed in **Table 7**. Notably, the three enhancements proposed in this study yielded positive effects on multiple facets of the original model. First, replacement of the C3 structure in MobileNetV3’s backbone network resulted in a notable mAP increase of 1.54%, reaching 80.51%. This enhancement also significantly reduced the footprint of the model by 37.6%, decreased the number of parameters by 39.6%, and boosted the frame rate by 3.42 frames per second. Subsequently, the addition of the Acmix module further improved the mAP and frame rate of the model, albeit not to the same extent as the inclusion of MobileNetV3 alone. Finally, the integration of the MetaAconc module into the model facilitated an adaptability to specific input data during training, culminating in an enhanced performance across various tasks. This configuration achieved an mAP of 80.78%, featuring 5,424,971 parameters, a model size of 20.9 MB, and a frame rate of 40.42 frames per second. Following network optimization, the mAP experienced a 0.59% improvement, with no changes in the total number of model parameters and detection speed. Compared with the original YOLOv5 model, the enhanced model demonstrated a 1.81% improvement in mAP, a substantial 22.88% reduction in model size, and a notable 25.45% decrease in the number of parameters. In conclusion, the method proposed in this study delivers a rapid high-accuracy detection performance across small and large scales. This meets the requirements for real-time detection while maintaining a compact model size.

**Table 7:** Impact of various enhancement strategies on model performance.

Model	mAP@0.5 (%)	Parameters	Model size (MB)	FPS
YOLOv5s	78.97	7,276,605	27.1	47.22
+Acmix	80.35	8,106,537	30.3	43.14
+MobileNetV3	80.51	4,395,327	16.9	50.64
+MetaAconc	80.18	47,098,541	27.2	49.64
+Acmix+MobileNetV3	80.19	5,412,267	20.8	39.34
+Acmix+MetaAconc	79.90	7,908,875	30.3	42.33
+MobileNetV3+MetaAconc	79.91	4,595,039	17.7	52.26
+Acmix+MobileNetV3+MetaAconc	80.78	5,424,971	20.9	40.42

### 3.7 Comparative experiments

In this study, we retrained several conventional network models to assess the performance differences between the improved models and their established counterparts. We employed a control-variable approach to ensure the accuracy of the computational results. Subsequently, we compared the detection results of the various network models using the same test dataset. The comparative results are presented in **Table 8**, highlighting the disparities in the mAP detection performance, model size, and detection speed. For multi-target kernel detection, our improved model achieved the highest recognition accuracy, surpassing the original YOLOv5s model. Specifically, it outperformed the YOLOv4_tiny (75.47%), EfficientNet_YOLOv3 (75.95%), MobileNetV1_YOLOv4 (73.77%), YOLOv3 (80.56%), and YOLOv4 (80.52%) models by 1.81%, 5.31%, 4.83%, 7.01%, 0.22%, and 0.26%, respectively. Concerning parameter count, our improved model stood out with only 5,424,971 parameters, which was significantly lower than the other comparison models. In terms of model size, our model’s footprint was merely 20.9 MB, making it the most compact, in stark contrast to the YOLOv4 model’s size of 244 MB and the YOLOv3 model’s size of 235 MB. Furthermore, our improved model achieved a detection frame rate of 40.42 frames per second, surpassing the YOLOv4 model by 7.77 frames per second and EfficientNet_YOLOV3 by 8.32 frames per second. In summary, our enhanced lightweight walnut detection model excels in recognition accuracy, boasts a compact model size, and demonstrates superior inference speed compared with its counterparts.

**Table 8:** Detection results of various target detection algorithms on walnut images.

Model	mAP@0.5 (%)	Parameters	Model size (MB)	FPS
YOLOv5s_Acmix_ MobileNetV3_MetaAconc (ours)	**80.78**	**5,424,971**	**20.9**	40.42
YOLOv5s	78.97	7,276,605	27.1	47.22
YOLOv4_tiny	75.47	6,056,606	22.4	**111.48**
EfficientNet_YOLOv3	75.95	10,776,233	60	32.1
MobileNetV1_YOLOv4	73.77	12,692,029	51.1	57.06
YOLOv3	80.56	61,949,149	235	48.23
YOLOv4	80.52	64,363,101	244	32.65

The bold values indicate the optimal values corresponding to the four groups of data: map@0.5(%)80.78 has the highest precision and is marked with bold; The number of parameters is 5,424,971, with the minimum marking thickness; Model size 20.9MB, minimum size; FPS11.48, the fastest detection speed.

**Figure 10:**
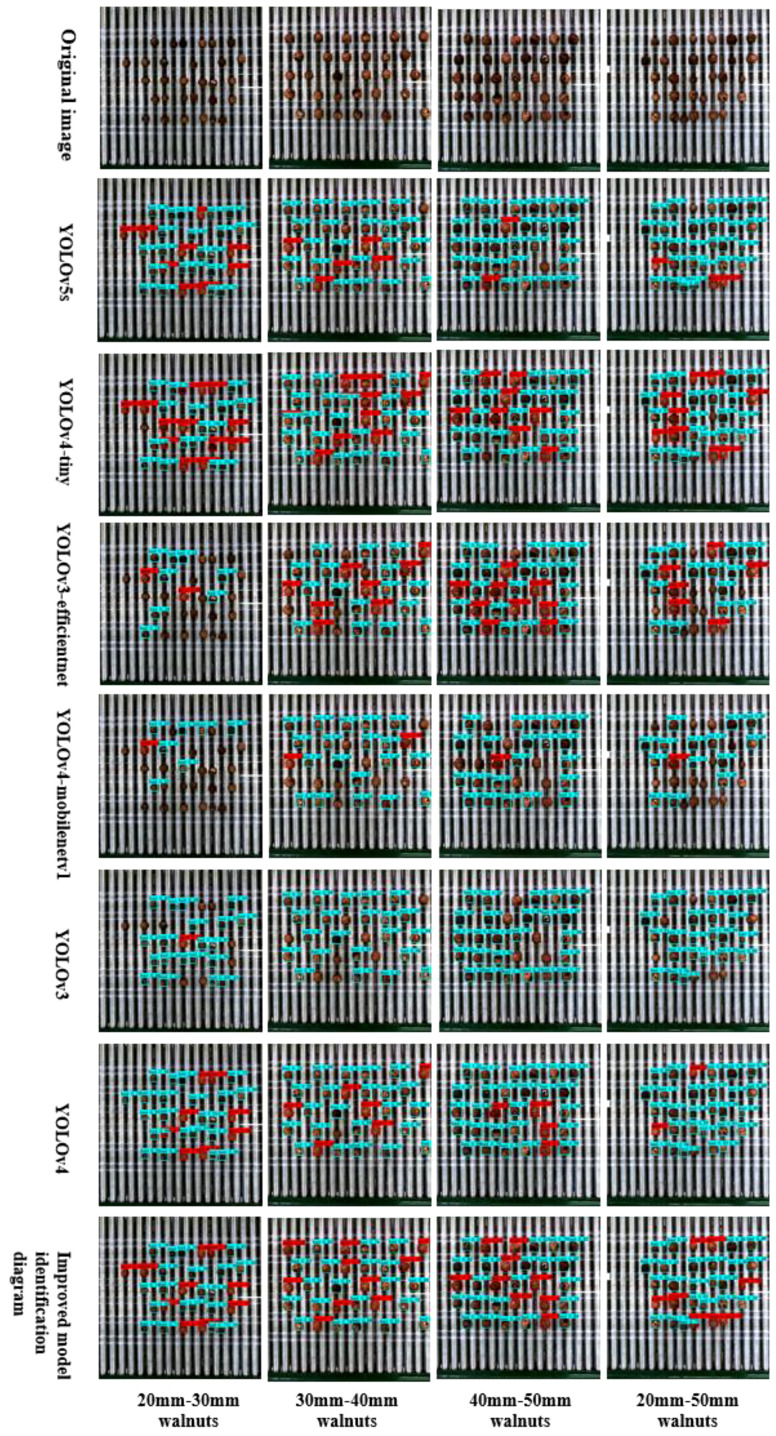
Detection performance of mainstream detection networks for good and defective walnuts of various sizes.

As shown in **Figure 10**, We selected images captured from the actual primary processing grading equipment to represent different sizes of walnuts, including small targets (20–30 mm), medium targets (30–40 mm), Each size category comprised 10 good and 20 bad fruits, with 10 each of black spots and broken fruits. Additionally, we included 30 walnuts ranging from 20 to 50 mm (comprising large, medium, and small sizes of 10 each), featuring 3 good and 7 bad fruits (4 with black spots and 3 broken fruits) for each size. Subsequently, we compared and examined the true results for each walnut size and category (**Table 9**). The experimental findings, in terms of the identification of good, bad, unchecked, and incorrectly detected walnuts, affirm the improved YOLOv5s_AMM model’s efficacy and precision in discerning good and bad walnuts across large, medium, and small targets. Remarkably, there were minimal instances of unchecked and incorrectly detected walnuts of different sizes. Notably, the detection of small target walnuts, characterized by a complex surface morphology and small size, poses a significant challenge. Although the YOLOv5s, YOLOv4_tiny, and YOLOv4 models exhibited relatively similar recognition results to the improved model, occasional cases of non-detection and incorrect detection were observed, underscoring the improved model’s superiority. Comparatively, the YOLOv4_tiny and EfficientNet_YOLOv3 models displayed slightly better results than the improved model, but with a notable increase in false detections and non-detections. Conversely, models such as YOLOv3 and the original YOLOv5s demonstrated ineffectiveness at detecting good fruits, with a significant number of non-detections and false detections. In conclusion, the enhanced YOLOv5s_AMM model consistently demonstrated its effectiveness and precision in identifying good and bad walnut fruits across varying sizes, as assessed by a composite set of criteria encompassing good and bad fruit identification and unchecked and incorrectly detected walnuts.

**Table 9:** Number of good and bad walnuts detected by mainstream target detection networks for various walnut sizes.

Model	20mm–30mm good/bad/ uninspected/ misinspected	30mm–40mm good/bad/ uninspected/ misinspected	40mm–50mm good/bad/ uninspected/ misinspected	20mm–50mm good/bad/ uninspected/ misinspected
YOLOv5s_Acmix_MobileNetV3_MetaAconc (ours)	**10/20/0/0**	**10/20/0/0**	**9/21/0/1**	**8/22/0/2**
YOLOv5s	9/21/0/1	5/23/2/3	2/27/1/8	3/26/1/4
YOLOv4_tiny	13/26/1/3	10/20/0/4	8/21/1/1	8/20/2/3
EfficientNet_YOLOv3	2/8/20/0	8/16/6/0	9/20/1/3	6/14/10/3
MobileNetV1_YOLOv4	1/7/22/2	2/19/9/3	1/21/8/1	1/14/15/1
YOLOv3	1/20/9/1	0/22/8/3	0/25/5/0	0/23/7/3
YOLOv4	8/22/0/2	6/23/1/3	4/24/0/5	2/27/1/5

The bold values represent the best case for each of these four sets of cases [best for good, bad, undetected, false (quantity) data in different sizes (20-30\30-40\40-50\20-50 \20-50).

## 4 Discussion

This study introduces a rapid non-destructive detection model designed to enhance the performance of the original YOLOv5s network for the detection of good and bad fruits within multi-target samples of dried walnuts. The dataset encompassed specimens that exhibited both desirable attributes and imperfections, including instances of black spotting and breakage. It is worth noting that extant research concerning walnuts predominantly centers on fresh green walnuts or kernels obtained from orchard trees, as shown in **Table 10**. Historical limitations have constrained access to extensive repositories of high-resolution imagery depicting good and bad dried walnuts, thereby constraining the scope of deep learning investigations in this domain. Recently, there has been a prevalent shift towards machine learning and convolutional neural networks in the context of kernel defect detection. The datasets used in related literature predominantly consist of single-object images captured within controlled laboratory environments or images featuring multiple object sets against the backdrop of orchard settings. In the interest of equitable assessment pertaining to image composition, network architecture, and detection efficacy within the chosen dataset, the findings presented in **Table 10** elucidate discernible enhancements in detection performance achieved through the deployment of various optimized network configurations relative to the original model. Consequently, these results underscore both the effectiveness and necessity of augmenting the detection capabilities of networks tasked with discerning multiple objects of diverse sizes.

**Table 10:** Recent research on target detection in walnut studies.

Objects	Networks	Dataset conditions	mAP	F1	Accuracy
Walnut foreign body (Rong et al., 2019)	Machine vision combines two different convolutional neural networks	Walnuts, natural foreign objects, and artificial foreign objects	—	—	95%
Pecan abscission, shell, and embryo area (Costa et al., 2021)	Machine vision combined with Mask-RCNN	Abscission, shell, and embryo areas in both small (young) and large (old) pecans at multiple growth stages	—	95.3% ~100%	—
Green walnut in natural environments (Fan et al., 2021)	Improved and faster R-CNN	Detection of green walnuts in natural environments (uniform light, uneven light, overlapping objects, shading, and varying target sizes)	97.71%	96.12%	—
Green walnut in natural environments (Hao et al., 2022)	Improved YOLOv3 (MobileNetV3)	Green walnuts on trees in the orchard (large targets, small targets, and backlighting conditions)	86.11%	—	—
English walnut kernel pericarp colour (Donis-Gonzalez et al., 2020)	Machine vision combined with a stepwise logistic regression method	English walnut kernels with different coloured peels	—	—	“Chandler” model (88.8%), seedling model (80.4%), and “Howard” model (75.1%)
Walnut impurities (Yu et al., 2023)	Improved YOLOv5 (Transformer and GhostNet)	Small impurities within walnut kernels	88.9%	—	—
Walnut fresh fruit (Zhang et al., 2016)	Machine vision combining hybrid features with the least squares support vector machines	Identification of fresh pecan fruits under natural scenes, considering downlight backlighting and branch shading	—	—	92.48%

In this study, we analyzed the experimental results obtained from the improved YOLOv5s_AMM detection model. The primary focus of this study was to address the challenge of discerning good and bad fruit images among multi-target walnuts of varying sizes. Moreover, we assessed the recognition performance of the improved model across walnut images with different dimensions. Within this analysis, we explored the impact of various attention mechanisms (**Table 11**) and the influence of different positions of improvement (**Table 6**) on the model’s recognition capabilities. Notably, the enhancements made to the original YOLOv5s model encompassed the incorporation of the Acmix structure, which introduces convolutional mixing, following the SPP layer. In addition, the activation function within the neck layer convolution was replaced with the MetaAconC activation function. These improvements were substantiated by the ablation (**Table 7**) and comparative experiments (**Table 8**). The experimental results presented in this study demonstrate the ability of the enhanced YOLOv5s_AMM detection model to swiftly and accurately identify good and bad walnuts within mixed images of dried walnuts, encompassing multiple targets of varying sizes. Furthermore, comparative experiments involving diverse improved modules and different typical target detection networks contribute to a comprehensive evaluation of the proficiency of the model in recognizing good and bad walnut fruits.

**Table 11:** Comparison of the effects of adding the attention mechanism at different positions.

Applied position	mAP0.5 (%)	Parameters	Model size (MB)	FPS
None	78.97	7,276,605	27.1	47.22
Position 1	80.35	8,106,537	30.3	43.14
Position 2	78.90	7,833,645	29.2	28.39
All	79.69	8,663,577	32.4	26.57

In essence, our proposed enhanced network exhibits improved detection performance, reduced model size, and accelerated inference speed when tasked with identifying mixed multi-target dried walnut fruits of varying sizes. This characteristic holds a significant promise for deployment in resource-constrained edge devices. In future research endeavours, we plan to prioritize the refinement of recognition accuracy and the model’s generalization capabilities. This will entail extending its applicability to encompass a broader spectrum of walnut variety recognitions. Subsequently, we aim to implement an improved model within the grading equipment used in the primary processing stages of walnuts. This deployment is envisaged to not only augment the value of walnut products but also enhance the efficiency of the walnut industry’s grading processes.

## 5 Conclusions and future work

This study focused on using photographs of walnuts collected after degreening, cleaning, and drying as the research dataset. In response to the distinctive visual attributes of walnuts within the primary processing context, we developed and implemented a method for detecting multiple good and bad walnut fruit targets. To support this investigation, we collected a substantial volume of multi-target walnut images, thereby constructing a corresponding dataset. To enhance the efficiency of the model while maintaining its lightweight architecture, we replaced the C3 network in the original YOLOv5s with MobileNetV3, resulting in an M3-Net network. Subsequently, we explored the impact of various attention mechanisms and improvement positions on the walnut images. Notably, the Acmix structure after the SPP layer was introduced, integrating attention and hybrid modules to capture both global and local image features. This strategic modification reduces network computational costs while augmenting performance on complex datasets. Furthermore, the MetaAconC activation function of the CBM module in the neck layer was replaced with an SiLU activation function from the original Conv layer. This adaptation improved the distribution of input-specific data for fine-tuning, thereby enhancing feature detection across various image scales. Additionally, we assessed the effectiveness of the model across the walnut images in varying proportions. Finally, we conducted a comprehensive examination of the different improvement modules applied to the detection of walnut datasets within the backbone and neck layers of the Ai model. The performance of different target detection networks on walnut datasets were further investigated. The results of these experiments successfully validated the performance enhancements achieved by our improved model.

The principal findings of this study are summarized as follows:

(1) Compared with other target detection models, our improved model exhibited superior performance across multiple metrics, including detection precision, model size, parameter size, and detection speed. Notably, our improved model achieved the highest accuracy, with an mAP of 80.78. Moreover, it boasted the smallest model size, measuring 20.9 MB, which was notably 11.7 times and 11.2 times smaller than the model sizes of conventional algorithms such as YOLOv4 and YOLOv3, respectively. Simultaneously, the model maintained a detection speed of 40.42 frames per second, aligning with the lightweight nature of the model suitable for rapid walnut detection scenarios and substantially outperforming the YOLOv4 and YOLOv3 models in terms of speed. These results underscore the success of the improved model in achieving greater recognition accuracy, a compact model size, and rapid performance.
(2) In practical applications, the enhanced model was employed to distinguish between good and bad fruits of multi-target walnuts within the test set. Ablation experiments were conducted to assess its performance, which resulted in an mAP of 80.78%. Compared with the original YOLOv5s model, our enhanced model exhibited an increase of 1.81% in mAP, a reduction of 22.88% in model size, and a decrease of 25.45% in parameter count, while maintaining a negligible difference in FPS. Additionally, experimental results involving walnut image detection with varying target sizes indicate improved precision and robustness.
(3) By leveraging the capabilities of the improved YOLOv5s_AMM model, which addresses the gap in detecting walnuts of different sizes after peeling and drying in the preliminary processing stage, we intend to apply it to the preliminary processing operations of walnut processing enterprises. Specifically, the model was employed for the detection and grading of good and bad walnut fruits after the peeling, washing, and drying stages. Our model offers distinct advantages, including a high recognition accuracy and compact model size.

## Data availability statement

The raw data supporting the conclusions of this article will be made available by the authors, without undue reservation.

## Author contributions

ZZ designed a lightweight model and trained the model. HZ walnut multi-target image acquisition; LL and YL lead many experiments and revisions to the text; ZL and HZ labeled walnut data set; XL to help build collection equipment and purchase materials; FZ and YZ received guidance and financial support for their experiments. All authors contributed to this article and approved the submitted version.

## Footnotes

1
https://openstd.samr.gov.cn/bzgk/gb/newGbInfo?hcno=11E65C73CF8B9E071CE76716628F2F80]
